# Supplementary material for: A public context with higher minority stress for LGBTQ* couples decreases the enjoyment of public displays of affection
Source: PLoS One. 2021 Nov 17;16(11):e0259102. doi: 10.1371/journal.pone.0259102 (PMC8598037; doi:10.1371/journal.pone.0259102)
Supplement: S1 Materials — (DOCX) [file pone.0259102.s001.docx]

**S1 Materials. Exploratory Measures in Study 1**

For exploratory reasons, we assessed a variety of other scales. Participants answered 12 items on self-stigmatization concerning their sexual identity *and the same 12 items with regard to their* gender identity on a scale from 1 (*totally disagree*) to 7 (*totally agree*) (Mohr & Kendra, 2011), four items on outness (Mohr & Kendra, 2011) with answers ranging from 1 (*does not know*) to 7 (*definitely knows and openly talked about*), seven items on social integration (Keyes, 1998) from 1 (*strongly disagree*) to 6 (*strongly agree*), seven items on campus climate (Rankin, 2005) from 1 (*strongly disagree*) to 4 (*strongly agree*), four question on academic success (average grade, self-assessment of performance, satisfaction with performance, and plans after universitary degree), one item on life satisfaction (Cheung & Lucas, 2014) ranging from 1 (*very dissatisfied*) to 10 (*very satisfied*) and one item on happiness (Cheung & Lucas, 2014) ranging from 1 (*not happy at all*) to 5 (*very happy*).

**References**

Cheung, F., & Lucas, R. E. (2014). Assessing the validity of single-item life satisfaction measures: Results from three large samples. *Quality of Life Research*, *23*(10), 2809-2818. https://doi.org/10.1007/s11136-014-0726-4

Keyes, C. L. M. (1998). Social well-being. *Social psychology quarterly*, *61*(2), 121-140. https://doi.org/10.2307/2787065

Mohr, J. J., & Kendra, M. S. (2011). Revision and extension of a multidimensional measure of sexual minority identity: The Lesbian, Gay, and Bisexual Identity Scale. *Journal of Counseling Psychology*, *58*(2), 234. https://doi.org/10.1037/a0022858

Rankin, S. R. (2005). Campus climates for sexual minorities. *New Directions for Student Services*, *2005*(111), 17-23. https://doi.org/10.1002/ss.170
