# Supplementary material for: A public context with higher minority stress for LGBTQ* couples decreases the enjoyment of public displays of affection
Source: PLoS One. 2021 Nov 17;16(11):e0259102. doi: 10.1371/journal.pone.0259102 (PMC8598037; doi:10.1371/journal.pone.0259102)
Supplement: S2 Materials — (DOCX) [file pone.0259102.s002.docx]

**S2 Materials. Exploratory Measures in Study 2**

For exploratory purposes, some of the scales from Study 1 were employed again in this study. Participants again answered questions on outness (Mohr & Kendra, 2011), social integration (Keyes, 1998), and life-satisfaction (Cheung & Lucas, 2014). In this study, self-stigmatization was assessed with an adapted version of the lesbian, gay, and bisexual identity scale (LGBIS; de Oliviera, Lopes, Costa, & Nogueira, 2012) which included 13 items with answers ranging from 1 (*absolutely disagree*) to 7 (*absolutely agree*). Self-control was newly added as an exploratory measure. For this, we used the short version of the self-control scale (SCS-K-D; Bertrams & Dickhäuser, 2009) which includes 13 items with answers ranging from 1 (*completely incorrect*) to 5 (*completely correct*). Campus climate was assessed with four items. An example would be, “I think the social climate of my university is open and friendly towards LGBTQ* individuals”, with answers ranging from 1 (*do not agree at all*) to 2 (*totally agree*). Academic success was assessed with seven questions. An example would be, “How would you asses your own performance compared to the average student?”, with answers ranging from 1 (*definitely below average*) to 7 (*definitely above average*). 0

**References**

Bertrams, A., & Dickhäuser, O. (2009). Messung dispositioneller Selbstkontroll-Kapazität: eine deutsche Adaptation der Kurzform der Self-Control Scale (SCS-K-D). *Diagnostica*, *55*(1), 2–10. https://doi.org/10.1026/0012-1924.55.1.2

Cheung, F., & Lucas, R. E. (2014). Assessing the validity of single-item life satisfaction measures: Results from three large samples. *Quality of Life Research*, *23*(10), 2809-2818. https://doi.org/10.1007/s11136-014-0726-4

de Oliveira, J. M., Lopes, D., Costa, C. G., & Nogueira, C. (2012). Lesbian, gay, and bisexual identity scale (LGBIS): Construct validation, sensitivity analyses and other psychometric properties. *Spanish Journal of Psychology*, *15*(1), 334–347. https://doi.org/10.5209/rev_.2012.v15.n1.37340

Keyes, C. L. M. (1998). Social well-being. *Social psychology quarterly*, *61*(2), 121-140. https://doi.org/10.2307/2787065

Mohr, J. J., & Kendra, M. S. (2011). Revision and extension of a multidimensional measure of sexual minority identity: The Lesbian, Gay, and Bisexual Identity Scale. *Journal of Counseling Psychology*, *58*(2), 234. https://doi.org/10.1037/a0022858
