# Supplementary material for: A public context with higher minority stress for LGBTQ* couples decreases the enjoyment of public displays of affection
Source: PLoS One. 2021 Nov 17;16(11):e0259102. doi: 10.1371/journal.pone.0259102 (PMC8598037; doi:10.1371/journal.pone.0259102)
Supplement: S3 Materials — (DOCX) [file pone.0259102.s003.docx]

**S3 Materials. Exploratory Measures in Study 3**

For exploratory purposes, we included a novel scale for sexual orientation which we adapted from the Gender-Inclusive Scale and the Sexual-Romantic Scale (Galupo, Lomash, & Mitchell, 2017). This scale aims at assessing sexual orientation on a continuum with all answers ranging from 0% attraction to 100% attraction.

For exploratory purposes we additionally presented participants with questions on their relationship satisfaction (if they indicated being in a relationship) and their sex drive. Relationship satisfaction was assessed with the German adaptation of the Relationship Assessment Scale (Hendrick, 1988; adapted by Hassebrauck (1991) and consisted of seven items. An example would be “I thoroughly love my partner” and answers ranged from 1 (*not at all*) to 7 (*very much*) for this item. Sex drive was assessed with the Trait Sex Drive Scale (TSDS; Weber, Reis, Frankenbach, & Friese, in preparation) and consisted of ten items. An example would be: “On a typical day: How often do you experience sexual desire?” Answers to all items ranged from 1 (*never*) to 5 (*very often*).

**References**

Galupo, M. P., Lomash, E., & Mitchell, R. C. (2017). “All of my lovers fit into this scale”: Sexual minority individuals’ responses to two novel measures of sexual orientation. *Journal of Homosexuality*, *64*(2), 145–165. https://doi.org/10.1080/00918369.2016.1174027

Hassebrauck, M. (1991). ZIP–Ein Instrumentarium zur Erfassung der Zufriedenheit in Paarbeziehungen. *Zeitschrift für Sozialpsychologie*, *22*(4), 256-259. Retrieved from https://psycnet.apa.org/record/1992-87357-001

Hendrick, S. S. (1988). A generic measure of relationship satisfaction. *Journal of Marriage and Family, 50*(1), 93-98. https://doi.org/10.2307/352430

Weber, M., Reis, D., Frankenbach, J., & Friese, M. (in preparation). Development and validation of the Trait Sex Drive Scale (TSDS). Unpublished manuscript.
