# Supplementary material for: A public context with higher minority stress for LGBTQ* couples decreases the enjoyment of public displays of affection
Source: PLoS One. 2021 Nov 17;16(11):e0259102. doi: 10.1371/journal.pone.0259102 (PMC8598037; doi:10.1371/journal.pone.0259102)
Supplement: S1 Table — (DOCX) [file pone.0259102.s004.docx]

**S4 Table. Locations of Participants in Study 3**

| Location | Number of participants | % |
| --- | --- | --- |
| Saarbrücken | 131 | 48.88 |
| Mainz | . 27 | 10.07 |
| Trier | .18 | 6.72 |
| Bielefeld | 9 | 3.36 |
| Heidelberg | 7 | 2.61 |
| Ulm | 7 | 2.61 |
| Köln | 6 | 2.24 |
| Bonn | 5 | 1.87 |
| Aachen | 5 | 1.87 |
| Lübeck | 4 | 1.49 |
| Berlin | 3 | 1.12 |
| Freiburg | 3 | 1.12 |
| Dortmund | 3 | 1.12 |
| Frankfurt | 3 | 1.12 |
| Eichstätt | 3 | 1.12 |
| Darmstadt | 2 | < 1 |
| Düsseldorf | 2 | < 1 |
| München | 2 | < 1 |
| Dresden | 2 | < 1 |
| Kaiserslautern | 2 | < 1 |
| Tübingen | 2 | < 1 |
| Erlangen/Nürnberg | 1 | < 1 |
| Kiel | 1 | < 1 |
| Gießen | 1 | < 1 |
| Fulda | 1 | < 1 |
| Landau | 1 | < 1 |
| Lüneburg | 1 | < 1 |
| Magdeburg | 1 | < 1 |
| Paderborn | 1 | < 1 |
| Regensburg | 1 | < 1 |
| Birkenfeld | 1 | < 1 |
| Würzburg | 1 | < 1 |
| Augsburg | 1 | < 1 |
| Duisburg | 1 | < 1 |
| Hamburg | 1 | < 1 |
| Koblenz | 1 | < 1 |
| Weiden | 1 | < 1 |
| Not specified | 6 | 2.24 |
| Total / *N* | 262 | 100.00 |
