# Supplementary material for: A public context with higher minority stress for LGBTQ* couples decreases the enjoyment of public displays of affection
Source: PLoS One. 2021 Nov 17;16(11):e0259102. doi: 10.1371/journal.pone.0259102 (PMC8598037; doi:10.1371/journal.pone.0259102)
Supplement: Attachment — Submitted filename: Revision Notes.docx [file pone.0259102.s005.docx]

# Revision notes, Manuscript PONE-D-21-11276

In these revision notes, we address the editor’s and the reviewer’s suggestions point-by-point. Our comments are marked with →. We start with the issues pointed out in the editorial letter, and then proceed to the reviewer’s additional comments. Additionally, we adjusted the word count, made minor linguistic improvements, updated the references, updated Figure 2, 3 and 4, and added a new discussion point (p. 36). A formal change concerns the name change of the first author. Due to the wedding of the first author, the name Hess was changed to Stammwitz.

We thank the editor and the reviewer for the time and thought they have invested in our work. The feedback we received helped us a lot to craft a much stronger contribution.

# Editor

Thank you for submitting your manuscript to PLOS ONE. After careful consideration, we feel that it has merit but does not fully meet PLOS ONE’s publication criteria as it currently stands. Therefore, we invite you to submit a revised version of the manuscript that addresses the points raised during the review process.

Given the rich review offered by this one Reviewer and the difficulty of finding a second Reviewer, I believe that authors can take advantage of the advice offered here to make their article suitable for publication.

When submitting your revision, we need you to address these additional requirements.

1. Please ensure that your manuscript meets PLOS ONE's style requirements, including those for file naming.

→ We checked all files according to style guidelines. Please do not hesitate to contact us if any style requirements are not met.

2. Please amend your manuscript to include your abstract after the title page.

→ The manuscript now included the abstract after the title page.

3. Your ethics statement should only appear in the Methods section of your manuscript. If your ethics statement is written in any section besides the Methods, please move it to the Methods section and delete it from any other section. Please ensure that your ethics statement is included in your manuscript, as the ethics statement entered into the online submission form will not be published alongside your manuscript.

→ We moved our ethics statement to the methods section.

# Reviewer 1

The paper aims to investigate whether minority stress (e.g., perceived discrimination) and physical displays of affection (PDA) vary across contexts (i.e., campus, city center, and private home – Study 3). It reports three studies conducted in Ireland and Germany among both cis-heterosexual and LGBTIQ+ university students. Notably, the last study relies on both quantitative and qualitative data.

Overall, I very much enjoyed reading this paper. The manuscript is clear, generally well written, and addresses an interesting topic. I particularly appreciate the use of a mixed-method approach in Study 3, which is a great strength of this manuscript. The findings of the qualitative analyses show a very interesting opposing/conflictual effect of context on PDA. I believe this finding is of particular relevance to the literature. I also appreciate that the authors focus on the context, which is often overlooked in the literature.

→ Thank you very much for the appreciation and your overall very positive evaluation of our paper.

Next, I list the issues I identified.

## Major:

1. The manuscript focuses on the concept of ‘minority stress (Meyer, 2003)’. This concept is, however, underdeveloped in the introduction. I would like the authors to develop this section further (e.g., by also referring to Hatzenbuehler research). This is particularly important because the authors mainly focus on one dimension of the model (i.e., distal stressors) without considering other forms of stressors (i.e., proximal stressors).

→ Thank you for this important theoretical addition. We now added information about proximal stressors to the paragraph dealing with minority stress. We also included references to Hatzenbuehler research (reference numbers 9 and 12). The new paragraph now reads (p. 3-4):

“According to Meyer’s minority stress theory (4), members of a minority group are (more) likely to face minority stress. An “underlying assumption” of the theory is that minority stress is both “unique” and “additive to general stressors that are experienced by all people, and therefore, stigmatized people require an adaptation effort above that required of similar others who are not stigmatized” (Meyer, 2003, p. 676). Meyer (2003) also suggested a distinction between distal and proximal stressors. Distal stressors are typically incidents that are objectively harmful and tied to the reaction of the outside world. They include a variety of behaviors, such as physical attacks, verbal (harassment), and para-verbal societal marginalization (e.g., a disapproving look or spitting on the floor). Proximal stressors are more subjective as they include individual perceptions and internalized stigmatization. Our research focuses mainly on distal stressors. However, Meyer pointed out that the proximal-distal distinction may be viewed as falling along a continuum with distal factors laying the groundwork for proximal (personal) stress. Also, regardless of any differentiation, the experience of minority stress typically has a negative impact on several dimensions of personal well-being and health (5–8). Minority stress seems to increase the risk of psychopathology and appears to have an overall negative effect on mental health (9). For example, minority stress (e.g., perceived discrimination based on sexual orientation) is associated with higher emotional distress (10) and higher levels of depressive symptoms and suicidal thoughts among LGBTQ* youth (11). For a review of minority stress as a social determinant of health, see (12).

So far, research investigating the effects of minority stressors has primarily focused on the individual, but it is easy to imagine that minority stress could also affect LGBTQ* couples. Indeed, a meta-analysis found that negative attitudes, judgments, or behaviors toward LGBTQ* individuals and couples were inversely associated with relationship functioning (13). Similarly, another meta-analysis reported that LGBTQ* minority stressors were negatively correlated with relationship well-being (14).”

2. The method used to group participants differs between Studies 1-2 (i.e., LGBTIQ+ vs. cis heterosexual) and Study 3 (same-sex vs. other sex partners). I do understand the author's reasoning, but I see it as problematic for different reasons. First, the authors mention in Study 3 “An important change compared to Study 1 and 2 was that each participant was asked to explicitly state if their (hypothetical) partner was of the same or the different sex” (p.22); however, on page 11 (Study 1) it is mentioned that “Participants indicated which gender male or female, their (hypothetical) partner has (would have).” Please clarify whether this question was also asked in Studies 1 and 2. If so, please indicate whether the results of Studies 1-2 replicate using the Study 3 classification method. I would like to know whether part of (as not all items were asked to all participants) the results of Study 3 replicate using Studies 1-2 classification method.

→ Thank you for pointing out this important methodological issue. Indeed, we asked similar but not identical questions in Study 1, 2, and 3.

In Study 1 and 2 the question read:
What gender does/would your partner have? [Female/Male/Other]
In Study 3 the question read:
My partner is/would be [same-sex/different-sex].

First, because of this similarity, we now refrain from calling the question on the relationship type of the participant’s imagination task an important change compared to Study 1 and 2. The new sentence now reads:

“Each participant was asked to explicitly state if their (hypothetical) partner was of the same or the different sex” (p. 20)

Second, because we did not ask participants whether their partner was different-sex or same-sex we cannot directly replicate the results of Study 3 with Study 1 and 2. However, given the information on their own and their partners sex and gender we can infer the relationship type. Please note, that this method is somewhat prone to errors. For example, when a participant indicated they identified as “other” (rather than as male or female) the relationship type could not be identified unambiguously. In the paper, we now included a paragraph in the limitation section addressing this point. The new paragraph reads:

“Finally, we used one method to group participants in Studies 1 and 2 and another in Study 3. Whereas Studies 1 and 2 focused on (self-assigned) LGBTQ* participants as the minority group, Study 3 examined participants who self-assigned themselves to being in a same-sex relationship. Because we asked similar questions in all three studies, we were able to conduct an exploratory analysis on the data from Studies 1 and 2 with the classification method used in Study 3 and to analyze the data from Study 3 with the method used in Studies 1 and 2. These analyses showed two things: First, when we employed the classification method from Study 3 in Studies 1 and 2, the size of the sample of participants in a same-sex relationship was too small for us to have confidence in the results (nsame-sex = 17 for Study 1 and nsame-sex = 47 for Study 2). Second, the results did replicate in that the significant results from Studies 1 and 2 remained significant when we changed the classification method. This also means we were not able to find an effect of context on PDA in Study 2, regardless of the method used to classify the two groups. We also transferred the original grouping method from Studies 1 and 2 to Study 3 (LGBTQ*/Hetero Cis instead of same-sex/different-sex). Again, the results remained the same with one exception: Whereas the type of relationship (with nsame-sex = 80 and ndifferent-sex = 188) had a significant main effect on displays of affection, Pillai’s trace F(2, 265) = 8.04, p < .001, sexual and gender identity (nLGBTQ* = 139 and nHetero/Cis = 129) did not, Pillai’s trace F(2, 265) = 1.88, p = .154. Meanwhile, the main effect of context on displays of affection , Pillai’s trace F(4, 263) = 144.15, p < .001, and the interaction between context and sexual and gender identity, Pillai’s trace F(4, 26) = 4.29, p = .002, remained significant.” (p. 35-36)

3. Second, I think that the method used in Study 3 is quite problematic for bisexual/pansexual people. In general, considering the high levels of identity denial that bisexual/pansexual people have to face (see also Maimon et al., 2019), asking them to clearly state the gender of their imagined partner (if this was the case) is a bit problematic. While this concern cannot be addressed at this stage of the research, I would like the author to not refer to LGBTIQ+ vs. heteronormative (or cis-heterosexual) relationships in the results of Study 3 (as some bisexual people were classified in the heterosexual group). As stated by the author, “this way of assigning does not conform with the actual terminology of LGBTQ* which includes bisexuals with no regard to their partner’s gender” (p, 19). Please instead refer to different vs. same-sex partners. Please also indicate how you classified the 25 participants who did not identify as male or female.

→ This is indeed an important point and we can now see that our wording was problematic before. We now refer to different vs. same-sex partners throughout Study 3 as this wording is indeed more precise and non-discriminatory to bisexual and pansexual people. We also included some research by Maimon on the issue of bisexual/pansexual identity denial and negation (reference number 41)

The new paragraph in the method section reads:

“Because our research focused on distal stressors, which are more pronounced when people are perceived as LGBTQ* by others, we separated participants into two groups on the basis of whether they answered our questions with a same-sex or a different-sex partner in mind. As Meyer (40) pointed out: If someone is perceived as gay or lesbian, they will be more likely to be confronted with minority stressors regardless of whether this perception is aligned with their actual sexual and gender identity (e.g., bisexual). As the terminology of LGBTQ* includes bisexuals with no regard to their partner’s gender, we henceforth changed the names of our groups from “sexual and gender identity” (LGBTQ* vs. Hetero/Cis) to “relationship type” (same-sex vs. different-sex). For example, a bisexual female person with a male partner (both cisgender) would most likely be in the minority group in Studies 1 and 2 because they self-identified as LGBTQ* but might not be in the minority group in Study 3 if they indicated they had a different-sex partner. However, we do point out that bisexual and pansexual people are, of course, a minority group and are frequently confronted with identity denial and erasure (41). We are also aware of the fact that it might have been more difficult for bisexual and pansexual participants to classify their imagined partner.” (p. 21-22)

Regarding the question how we classified the 25 participants who did not identify as male or female: These participants also indicated if their partner was different or same-sex. If they had answered this question, they were classified according to their answer.

## Minor:

4. Please indicate the year of data collection and (if possible) the cities in which Studies 1&2 data were collected. Given that this article focuses on the ‘context,’ I think this information is of particular importance.

→ Thank you for bringing up this important point. Study 1 was conducted in Limerick (Ireland) in 2018. Study 2 was conducted in Saarbrücken (Germany) in 2018. Study 3 was conducted in Saarbrücken (Germany) in 2019. Kindly note that Study 3 included participants located in various German cities although more participants were recruited in Saarbrücken (*n* = 121) than in any other city. A distribution of participants’ locations can now be found in the supplemental material S4. This new information now reads:

“Study 3 (*N_total_* = 268, *n_same-sex_* = 80) was conducted in Saarbrücken, Germany in 2019. In contrast to Studies 1 and 2, participants were recruited not only at the respective university and city but nationwide. A distribution of participants’ locations can be found in Supplemental Material S4.” (p. 19)”

We also added the cities and the year of data collection to the respective sections:

“Study 1 was conducted in Limerick, Ireland in 2018.” (p. 9)
“Study 2 was a replication of Study 1 aimed at obtaining a larger sample size. It was conducted in Saarbrücken, Germany in 2018.” (p. 15)
“Study 3 (*N_total_* = 268, *n_same-sex_* = 80) was conducted in Saarbrücken, Germany in 2019.” (p. 19)

5. I would prefer to see the descriptive tables after the measure section. Please also add the p-values (for the correlations) in the tables.

→ The descriptive Tables 1, 2, 3 and 4 can now be found at the end of each of the respective methods section. P-values are now added to the correlation tables.

6. The term ‘sexual identity’ is used to refer to the difference between the two groups (LGBTQ+ vs. cis heterosexual). However, heterosexual trans people in Studies 1 and 2 were included in the LGBTQ+ sample, right? This is, thus, not consistent with the terminology (since their sexual identity is ‘heterosexual’). Please address this concern (e.g., by referring to sexual and gender identity).

→ Thank you for bringing up this important topic. Indeed, heterosexual trans people were included in the sample; we now referred to “sexual and gender identity” when differentiating between the groups LGBTQ* and Hetero/Cis in Study 1 and 2. Since we now use the terms same-sex and different-sex in Study 3, we use the term “relationship type” to refer to this distinction.

7. Study 3 is presented as a higher-powered study. Please revise this claim as the LBGTQ+ sample is not larger than in Study 2. Further, the design is quite unbalanced, which might impact some of the interactions.

→ We refrained from saying that Study 3 is higher powered. The new sentence/paragraph now reads:

“Study 3 (*N_total_* = 268, *N_same-sex_* = 80) was conducted in Saarbrücken, Germany in 2019” (p. 19)

8. On page 19, it is stated that “the survey was completed by N = 268 university students at more than 30 different German universities with NLGBTQ* = 80 and NHetero/Cis = 129”. The numbers don’t add up. Please clarify why (I might have missed something).

→ We are sorry for this misunderstanding. The number *n* = 80 refers to the participants with a same-sex partner. The number *n* = 128 refers to the number of participants who indicated they were heterosexual and cisgender. The missing *n* = 59 participants are LGBTQ* but answered questions on PDA with a different-sex partner in mind. Since we have corrected our imprecise wording of the group LGBTQ* to same-sex, the paragraph should be a bit clearer now. The new section now reads:

“The survey was completed by *N* = 268 university students at more than 30 different German universities with *n_same-sex_* = 80,  *n_different-sex_* = 188, *n_LGBTQ*_* = 139 and *n_Hetero/Cis_* = 129.“ (p. 21)

9. The result section of Study 3 (quantitative part in particular) is quite long; maybe there is an option to condense part of it (e.g., by referring more to the figure/Table).

→ We now shortened the quantitative part from 1316 words to 1256 words and referred more to the tables and figures.

10. In the qualitative part of Study 3, it is mentioned that “some participants answered the initial question on why their PDA was more frequent and enjoyable in one context by explaining why their PDA was less frequent and enjoyable in the other context.” (p.29). This also indicates a possible order effect. I would be curious to see whether the quantitative results were impacted by the question order (e.g., whether one context was asked before another).

→ The order of questions on context was randomized and thus we are confident that any order effect should cancel out across participants.

11. On page 30, it is stated that “Moreover, while a context with increased minority stress does not seem to reduce the frequency in which LGBTQ*couples show affection (Study 3), it does impair their emotional affect during these affection displays…”. I might be mistaken, but is it not Study 2?

→ Study 3 showed that PDA frequency for same-sex couples was not impaired in a more harmful context while PDA enjoyment was. Study 1 and 2 did not include measures for PDA frequency. Study 1 showed that PDA enjoyment was lower in a more harmful context, a result that did replicate in Study 3 but not in Study 2. We adapted our wording in the paragraph which now reads:

“Our results show that LGBTQ* minority stress differed substantially between the campus and the city contexts in all three studies (supporting Hypothesis 1). This strongly indicates that minority stress is indeed sensitive to context (Research Question 1). Moreover, a context with increased minority stress seems to impair same-sex couples’ enjoyment during displays of affection, meaning they enjoy PDA less in more harmful public contexts (Studies 1 and 3). Meanwhile, such a harmful effect could not be found for the frequency in which same-sex couples show affection (Study 3, partly supporting Hypothesis 3). These findings indicate that the perception of minority stress translates at least partly into couple behavior (Research Question 2).“ (p. 32-33)

12. I sometimes felt like the discussion could be more integrated with the current literature (and what future research can learn from your findings).

→  We included more current literature in the discussion (Hocker et al., 2021; LeBlanc, 2020; Neilands et al. 2020). The new section now reads:

“In future research, a study could investigate the perception of LGBTQ* minority stress and PDA while the participants are actually present in the respective context. Alternatively, the descriptions of the chosen contexts could be augmented with more information and pictures to ensure that participants think of the same places while answering the questions hypothetically. Also, current research has begun to explore couple-level minority stress by using a dyadic sample of couples instead of individual participants (45)(21). This change may help disentangle the effects of individual and couple-level minority stress and provide an even better understanding of the matter of open displays of relationship behavior. For a scale that specifically measures couple-level minority stress, see Neilands et al. (46).” (p. 36)

Reference:

9.	Hatzenbuehler ML. How does sexual minority stigma “get under the skin”? A psychological mediation framework. Psychol Bull. 2009;135(5):707–30.

12.	Hatzenbuehler ML, Pachankis JE. Stigma and Minority Stress as Social Determinants of Health Among Lesbian, Gay, Bisexual, and Transgender Youth: Research Evidence and Clinical Implications. Pediatr Clin North Am. 2016 Oct;63(6):985–97.

21.	Hocker L, Kline K, Totenhagen CJ, Randall AK. Hold my hand: Associations between minority stress, commitment, and PDA for same-gender couples. Journal of Social and Personal Relationships [Internet]. 2021 May 31; Available from: https://doi.org/10.1177/02654075211020501

41.	Maimon M, Sanchez DT, Albuja AF, Howansky K. Bisexual identity denial and health: Exploring the role of societal meta-perceptions and belonging threats among bisexual adults. Self and Identity. 20(3):1–13.

45.	LeBlanc AJ, Frost DM. Couple-Level Minority Stress and Mental Health among People in Same-Sex Relationships: Extending Minority Stress Theory. Society and Mental Health [Internet]. 2020;10(3). Available from: https://doi.org/10.1177/2156869319884724

46.	Neilands TB, LeBlanc AJ, Frost DM, Bowen K, Sullivan PS, Hoff CC, et al. Measuring a New Stress Domain: Validation of the Couple-Level Minority Stress Scale. Arch Sex Behav. 2020 Jan;49(1):249–65.
