# Supplementary material for: A public context with higher minority stress for LGBTQ* couples decreases the enjoyment of public displays of affection
Source: PLoS One. 2021 Nov 17;16(11):e0259102. doi: 10.1371/journal.pone.0259102 (PMC8598037; doi:10.1371/journal.pone.0259102)
Supplement: Attachment — Submitted filename: Revision Notes 2.docx [file pone.0259102.s006.docx]

# Revision notes 2, Manuscript PONE-D-21-11276

In these revision notes, we address the editor’s suggestions point-by-point. Our comments are marked with →. There were two formal changes. First. the name change of the first author Hess was changed to Stammwitz. Second, the second author has a shared first authorship and is corresponding author.

We thank the editor and the reviewer for the time and thought they have invested in our work. The feedback we received helped us a lot to craft a much stronger contribution.

# Reviewer #1

I appreciate that the authors addressed my comments thoroughly. I am, in general, very happy with the revisions. I, however, still have a few minor concerns:

*→ Thank you very much for this positive feedback. We are happy that we were able to address your comments in detail.*

1. The authors did a great job in developing the ‘minority stress model’ in the introduction. On a minor note, the authors might consider splitting the new paragraph on p.4 into two (e.g., splitting at lines 36-37).

*→ We now splitted the paragraph on page 4 at lines 32-33, in order to differentiate between the paragraph dealing with distal and proximal stressors from the paragraph dealing with the negative consequences of experiencing minority stress.*

2. The description of the method used to group participants is much clearer now. The authors mention that the question in Studies 1-2 read: “What gender does/would your partner have? [Fem ale/Male/Other]”. However, in the text, it is mentioned that “Participants indicated which gender male or female, their (hypothetical) partner has.” Please revise the text to mention that participants could also indicate that they had a partner who did not fall into the gender binarity (i.e., the ‘other’ response category).

*→ On page12 ll. 235-236, we now included the other category in this information:*

*„Participants indicated the gender of their (hypothetical) partner (female/male/other).“*

3. I was also happy to see the new analyses using a different method to group participants in the discussion section. If the authors are short on the word limit (or want to shorten their discussion section), they might consider moving these findings to the supplementary material and mentioning them in the main text (or in a footnote).

*→ We are glad that the reviewer liked our addition about the grouping information. We decided to retain this addition in the limitation section, because footnotes are not an formal* *option at the journal and we think the information is worth to be mentioned in the main manuscript.*

4. Finally, I thank the authors for mentioning the limitations of the study regarding bi- and pansexual participants. In general, I like this addition. However, I still find the sentence “because our research focused on distal stressors, which are more pronounced when people are perceived as LGBTQ* by other […]” problematic. Indeed, if people are genuinely perceived as LGBTQ*, then this should not be depending on the sex of their partner (i.e., bisexual people should be considered LGBTQ* regardless of the sex of their partner). I would therefore suggest revising this sentence to, for instance: “because our research focused on distal stressors, which are more pronounced when people are with a same-sex vs. different-sex partner […]”.

*→ We now changed the sentence on p. 22 ll. 467-468 to:*

*„Because our research focused on distal stressors, which are more pronounced when people are with a same-sex vs. different-sex partner,...“*
